# Inhibition of G9a Histone Methyltransferase Converts Bone Marrow Mesenchymal Stem Cells to Cardiac Competent Progenitors

**DOI:** 10.1155/2015/270428

**Published:** 2015-05-21

**Authors:** Jinpu Yang, Keerat Kaur, Li Lin Ong, Carol A. Eisenberg, Leonard M. Eisenberg

**Affiliations:** New York Medical College/Westchester Medical Center Stem Cell Laboratory, Departments of Physiology and Medicine, New York Medical College, Valhalla, NY 10595, USA

## Abstract

The G9a histone methyltransferase inhibitor BIX01294 was examined for its ability to expand the cardiac capacity of bone marrow cells. Inhibition of G9a histone methyltransferase by gene specific knockdown or BIX01294 treatment was sufficient to induce expression of precardiac markers *Mesp1* and *brachyury* in bone marrow cells. BIX01294 treatment also allowed bone marrow mesenchymal stem cells (MSCs) to express the cardiac transcription factors *Nkx2.5*, *GATA4*, and *myocardin* when subsequently exposed to the cardiogenic stimulating factor Wnt11. Incubation of BIX01294-treated MSCs with cardiac conditioned media provoked formation of phase bright cells that exhibited a morphology and molecular profile resembling similar cells that normally form from cultured atrial tissue. Subsequent aggregation and differentiation of BIX01294-induced, MSC-derived phase bright cells provoked their cardiomyogenesis. This latter outcome was indicated by their widespread expression of the primary sarcomeric proteins muscle *α*-actinin and titin. MSC-derived cultures that were not initially treated with BIX01294 exhibited neither a commensurate burst of phase bright cells nor stimulation of sarcomeric protein expression. Collectively, these data indicate that BIX01294 has utility as a pharmacological agent that could enhance the ability of an abundant and accessible stem cell population to regenerate new myocytes for cardiac repair.

## 1. Introduction

The adult heart contains endogenous stem cells that contribute to new myocyte formation during normal homeostasis and in response to injury [1–3]. A common way to isolate these cardiac progenitor cells (CPCs) is by extended culture of explanted heart tissue, where CPCs become identifiable as small round phase bright cells (PBCs) that emerge on top of an underlying adherent cell layer [4–7]. The use of endogenous CPCs for treating human patients requires heart biopsies, which may not be an efficacious protocol for severely diseased individuals. Thus, there is the need to develop new and/or improve upon existing stem cell sources for cardiac therapies.

The abundance and accessibility of stem cells within the bone marrow have attracted considerable research attention on the potential value of this tissue for cardiac repair [8–11]. Although the differentiation potential of bone marrow stem cells extends beyond the cell types that reside within this tissue, the intrinsic capacities of these cells to generate cardiomyocytes appear to be low and far less than produced from embryonic stem cells (ESCs) or induced pluripotent stem cells (iPSCs) [12–15]. Since differentiation of pluripotent cells into individual cell types is difficult to control and both ESCs and iPSCs have a tendency to form tumors when introduced into adult tissues [16–18], we investigated whether the differentiation potential of bone marrow cells could be broadened without making the cells pluripotent. The shared mesodermal origin of bone marrow and heart suggested that bone marrow stem cells may be able to obtain cardiopotency without approaching a pluripotent phenotype. In a previous study [19], we reported that treatment of nondifferentiated bone marrow cells with the G9a histone methyltransferase (HMTase) inhibitor BIX01294 induced expression of precardiac marker genes and allowed these cells to differentiate in response to the cardiogenic stimulus Wnt11. To follow up those initial findings, we examined the effect that direct suppression of G9a HMTase by gene knockdown would have on bone marrow cell phenotype and explored whether inhibition of this enzyme would enhance the cardiac competency of mesenchymal stem cells (MSCs), which are the most abundant and readily isolated stem cell population in the bone marrow. The new data in this report provide further evidence that G9a HMTase is an effective target for enhancing bone marrow cell cardiocompetency, as inhibition of this enzyme provokes bone marrow MSCs to exhibit a similar phenotype and cardiac potential exhibited by endogenous stem cells of the adult heart.

## 2. Material and Methods

### 2.1. Isolation and Culture of Bone Marrow MSCs

Animal protocols were approved by the Institutional Animal Care and Use Committee at New York Medical College. Bone marrow was harvested from femurs collected from 8–12 wk C57BL/6 mice, as described [19]. Standard procedures were used to obtain MSCs from bone marrow [21–23]. Disaggregated bone marrow cells were resuspended at 10^6^ cells/mL in Iscove's Modified Dulbecco's Medium (IMDM) plus 20% FBS. After 4 hrs, cells unattached to the culture plastic were removed. The remaining cell layer was cultured and then split just before becoming confluent and repassaged. Cultures were harvested at passage 2, just prior to reaching confluency, for use in experimentation.

To begin treatment, MSCs were serum-starved overnight in IMDM and then cultured in fresh 10% FBS/IMDM in the presence or absence of various doses of BIX01294, CHIR99021, dorsomorphin, IWP4, 1,5-naphthyridine pyrazole derivative-19 (Alk5 inhibitor, Stemgent), 3-bromo-7-nitroindazole, trichostatin A (Cayman Chemical), SP600125, PD173074, and/or PNU74654 (Tocris Bioscience). Two days later, cells were either harvested for RNA or cultured with fresh medium consisting of 10% FBS/IMDM, plus or minus Wnt11, for seven days. Wnt11 was supplied as a 50% dilution of conditioned medium from Wnt11-secreting cells, as described [24], with levels of the protein verified by immunoblotting. Immunofluorescent staining of the cells was carried out following their replating into Lab-Tek 8-well chamber slides (Nunc) or after being cytospun onto histology slides [25, 26].

### 2.2. Small Hairpin RNA (shRNA) Knockdown

Knockdown of G9a HMTase was carried out using gene-specific shRNA plasmids GI540026 and GI540029 (from OriGene), which are denoted in this study as shRNA676 and shRNA3291 in reference to the initial nucleotide position of the G9a sequence recognized by the shRNAs. A control scrambled shRNA construct and empty HuSH cloning vector, which are provided by the manufacturer, served as negative controls. Long-term cultures of bone marrow cells [19] were transfected with plasmid DNA using GeneCellin Transfection Reagent (BioCellChallenge). Cells taking up the plasmids were selected under 1 *μ*M puromycin, with multiple cultures per group assayed for protein and gene expression.

### 2.3. Atrial Cultures and Generation of Cardiac Conditioned Medium (CCM)

Cardiac tissue was harvested from 8–12 wk C57BL/6 mice. Excised atria were minced into 1 mm^3^ fragments, washed with PBS, and digested with trypsin for 10 min, before plating on gelatin coated dishes for 14 days in IMDM containing 20% FBS plus Pen/Strep. Culture medium was collected twice a week, filtered through 0.2 *μ*m polyethersulfone membranes, and stored at −20°C as CCM. To minimize batch-to-batch variations, CCM were collected from multiple batches of atrial cultures and thoroughly mixed prior the filtration.

### 2.4. Formation, Harvesting, and Culturing of Phase Bright Cells

Bone marrow-derived MSCs were cultured in the absence or presence of 8 *μ*M BIX01294 for 48 hours. Subsequently, cells were cultured in fresh IMDM containing 10% FBS, 50% CCM for 2 weeks, with media changed twice weekly. Thereafter, cultures were incubated with 50% CCM, 1 × 10^−7^ M dexamethasone, 10% FBS/IMDM for an additional 2 weeks. During this latter period, unattached cells were periodically collected by media removal and then lightly adherent cells were detached from the cultures following exposure to 0.5 mM EDTA at room temperature for 2 minutes. Cells released by the EDTA were combined with the initial cell isolates as a pooled population of low adherent cells. Visual inspection verified that the pooled isolates comprised the PBC population of the cultures. Collected PBCs were aggregated by hanging drop for 4 days before transfer to tissue culture dishes for additional 4 weeks in CCM diluted 1 : 1 in 10% FBS/IMDM.

### 2.5. RNA Isolation and PCR Amplification

Total RNA was obtained with Quick-RNA MiniPrep kits (Zymo Research) and reverse-transcribed using Moloney murine leukemia virus reverse transcriptase (Promega). Comparative quantitative real-time PCR (qPCR) was performed using the Perfecta SYBR green FastMix Rox qPCR master mix (Quanta BioSciences), as described [19, 27]. Expression levels of phenotypic marker genes were normalized to* GAPDH* expression and calculated by the ΔΔCt method. Statistical differences were compared by unpaired Student's *t*-tests using the InStat statistical application (GraphPad Software). Significance was defined as *p* < 0.05, with error bars corresponding to standard error of the mean.

### 2.6. Immunofluorescent Staining

Immunofluorescent labeling was performed as previously described [19, 28, 29]. Methylation of histone 3 lysine 9 (H3K9) was assessed using rabbit anti-dimethylated H3K9 antibody (4658P, Cell Signaling Technology), following cell fixation with formalin, permeabilization for 10 min with 0.25% Triton-X100/PBS, and overnight block with 10% goat serum/PBS at 4°C. For Islet1 staining, cells were fixed for 10 minutes with formalin, followed by Dent's fixation for 5 minutes, and then permeabilized with 0.3% triton/10% BSA/PBS. Mouse anti-Islet1 (39.3F7, Developmental Studies Hybridoma Bank; DSHB) was applied after blocking overnight with 1% BSA/0.3 M glycine/PBS. Staining with anti-muscle *α*-actinin (EA-53, Sigma), desmin (D8281, Sigma), titin (9D10, DSHB), connexin40 (Cx40, 36-5000, Life Technologies), Cx43 (13-8300, Life Technologies), CD90 (553016, BD Pharmingen) and Sca-1 (16-598, eBioscience) antibodies followed methanol or paraformaldehyde fixation for 10 minutes, multiple PBS washes, and overnight incubation with 5% BSA/PBS blocking solution. DyLight 488 conjugated secondary antibody (Jackson ImmunoResearch) was used to label bound primary antibody, which was acquired as grey signal when imaged on the Zeiss LSM 710 confocal microscope using the associated ZEN imaging software (Zeiss). After processing cell samples for immunostaining, cells were counterstained with 4′,6′-diamidino-2-phenyindole (DAPI; Life Technologies) to identify nuclei, which was captured according to its blue fluorescence. To enhance the perception of DAPI signal in the digital images, a fraction of the blue signal was outputted to the green channel using Image J software (http://imagej.nih.gov/ij/).

### 2.7. Immunoblotting

Protein was extracted from bone marrow cells by lysis in RIPA buffer (50 mM Tris–HCl, pH 7.5, 150 mM NaCl, 1% sodium deoxycholate, 0.1% sodium dodecyl sulfate, and 1% Triton X-100) containing Mammalian ProteaseArrest protease inhibitors (G-Biosciences). Total protein was separated on polyacrylamide gels, transferred to polyvinyl difluoride membrane, and incubated with rabbit antibodies specific to total histone H3 (Millipore) dimethylated-H3K9, G9a HMTase (17-10046, Cell Signaling Technology), or GAPDH (ab8245, Abcam). Proteins were detected using alkaline phosphatase-coupled anti-rabbit IgG antibody (Promega) and PhosphaGLO Reserve AP Substrate (KPL, Inc.).

## 3. Results

### 3.1. Knockdown of G9a HMTase Promotes Precardiac Gene Expression

**Figure 1:**
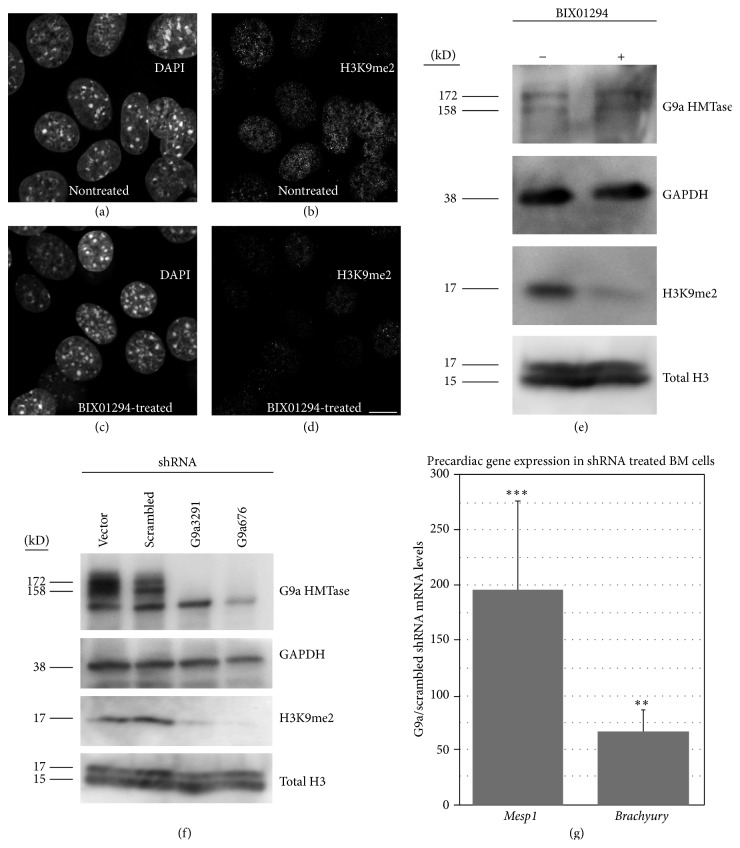
Effect of G9a HMTase inhibition on bone marrow cells. (a–d) Fluorescent staining of nontreated and BIX01294-treated bone marrow stem cells with DAPI for labeling all nuclei and antibody specific for dimethylated form of histone H3 at lysine 9 (H3K9). (e) Immunoblot of protein isolated from nontreated and BIX01294-treated bone marrow stem cells. Data from panels (a)–(e) demonstrate that methylation of H3K9 is reduced upon exposure to BIX01294. Blotting for GAPDH and total histone H3 verified equal amounts of protein were added for each sample. (f) Immunoblot showing that knockdown of G9a HMTase expression using either of two gene-specific shRNAs (G9a676 and G9a3291) caused decreased H3K9 dimethylation, in comparison to cultures transfected with scrambled shRNA or the empty vector. Blotting for GAPDH and total histone H3 served as controls. For all blots, side bars indicate the molecular weight of the specific proteins detected, including the 172 and 158 kD bands that correspond to the two known splice variants of G9a HMTase: G9a-L, G9a-S [20]. (g) Real-time qPCR analysis showing that G9a HMTase specific knockdown in bone marrow stem cells upregulated* Mesp1* and* brachyury* mRNA expression, as compared to scrambled shRNA controls, which is consistent with results obtained with BIX01294 treatments. ^*∗∗∗*^ *p* < 0.001; ^*∗∗*^ *p* < 0.005.

BIX01294 is a selective inhibitor of G9a HMTase [30–32], which is a methylating enzyme that targets lysine 9 on histone H3 (H3K9) [20, 32, 33]. As our previous studies had indicated that BIX01294 treatments induce expression of precardiac progenitor markers in bone marrow cells, we sought to verify that this phenotypic change was due to the inhibition of G9a HMTase. Long-term bone marrow cultures that were exposed to BIX01294 for 48 hrs showed a marked decrease in H3K9 methylation (Figures 1(a)–1(e)) without alteration of total histone H3 levels (Figure 1(e)). G9a HMTase levels were also unaffected by BIX01294 (Figure 1(e)), which is in accordance with the mechanism of BIX01294 interference with G9a HMTase activity being due to its competitive binding to the enzyme's substrate binding site [30]. Reduction of H3K9 methylation in response to BIX01294 was comparable to the effect observed from the direct inhibition of G9a HMTase expression by gene knockdown (Figure 1(f)). For the knockdown of G9a HMTase, two distinct gene-specific shRNAs were employed, which both suppressed production of G9a HMTase protein (Figure 1(f)). Both G9a HMTase-specific shRNAs greatly reduced methylation of H3K9, without affecting levels of histone H3 protein (Figure 1(f)). In contrast, neither scrambled shRNA nor empty vector reduced G9a HMTase expression or H3K9 methylation (Figure 1(f)). When G9a HMTase was knocked down with the gene-specific shRNAs, expression of the precardiac markers* Mesp1* and* brachyury* was induced in bone marrow cells (Figure 1(g)), which is consistent with our previous results showing induction of these precardiac genes in bone marrow cells in response to BIX01294 [19]. Thus, treatments that reduce G9a HMTase activity can provoke bone marrow cells to exhibit molecular markers that are characteristic of precardiac mesodermal cells of the early embryo.

### 3.2. Response of Bone Marrow MSCs to BIX01294

**Figure 2:**
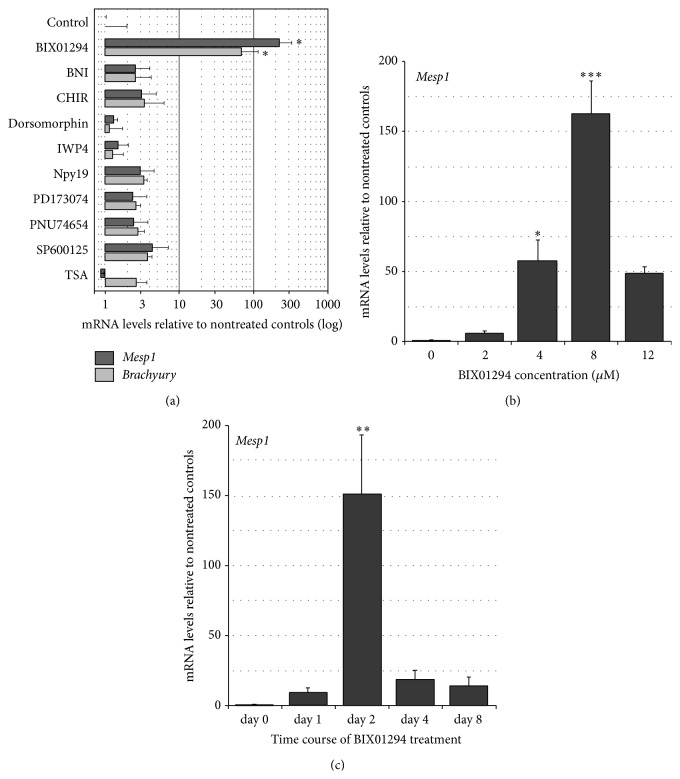
MSCs express precardiac markers in response to BIX01294. (a) MSCs were exposed to multiple compounds over a range of concentrations, with RNA harvested and analyzed by qPCR. Chart summarizes results with optimized doses of inhibitors specific for G9a HMTase (BIX01294), nitric oxide synthase (3-bromo-7-nitroindazole; BNI), GSK3*β* (CHIR99021), BMP (dorsomorphin), Wnt (IWP4), TGF*β* (1,5-naphthyridine pyrazole derivative-19; NPy19), FGF (PD173074), *β*-catenin (PNU74654) c-Jun N-terminal kinase (SP600125), and histone deacetylase (trichostatin A; TSA). Only BIX01294 was able to significantly enhance* Mesp1* and* brachyury* expression over control levels. Note that the relative levels of gene expression are shown in log scale. (b) Parallel cultures of MSCs treated for 48 hrs with various concentrations of BIX01294 and assayed for* Mesp1* gene expression using real-time qPCR. MSCs exhibited the highest levels of* Mesp1* expression when exposed to 8 *μ*M BIX01294. (c) MSCs exposed to 8 *μ*M BIX01294 for up to 8 days and examined for* Mesp1* gene expression, with optimal responses obtained at 48-hour incubation. ^*∗∗∗*^ *p* < 0.001; ^*∗∗*^ *p* < 0.005; ^*∗*^ *p* < 0.01.

Having established that BIX01294 can induce expression of precardiac phenotypic markers in heterogeneous bone marrow cultures, we investigated whether this drug would have greater efficacy in promoting cardiocompetency if the starting population was further enriched for stem cells. Thus, we investigated the responses of MSCs, which are the most abundant and readily isolated stem cell population in the bone marrow, but possess a limited cardiac potential. Because we did not assume that MSCs would behave identically as did the long-term bone marrow cultures to BIX01294, we extensively examined this drug's capabilities in promoting precardiac gene expression in MSCs. To determine whether BIX01294 possesses unique capacities in broadening MSC cell phenotypic potential, these experiments were done in parallel with treatments with other pharmacological reagents that, like BIX01294, were shown to have utility for assisting the generation and/or maintenance of pluripotent stem cells, but only as part of a compound treatment [34–40]. Specifically, we investigated the responses of MSCs to reagents that inhibit nitric oxide synthase (3-bromo-7-nitroindazole; BNI), GSK3*β* (CHIR99021), BMP (dorsomorphin), Wnt (IWP4), TGF*β* (1,5-naphthyridine pyrazole derivative-19; NPy19), FGF (PD173074), *β*-catenin (PNU74654), c-Jun N-terminal kinase (SP600125), and histone deacetylase (trichostatin A; TSA) activities. MSCs were treated with these various reagents or BIX01294 over a range of doses and exposure times and subsequently analyzed by real-time quantitative (q)PCR for expression of the transcription factors* Mesp1* and* brachyury*, which identify precardiac mesodermal progenitor cells within the early embryo [41–44]. In response to an optimum dose of BIX01294, MSCs produced 195- and 98-fold increases of* Mesp1* and* brachyury* gene expression, respectively, as compared to nontreated controls (Figure 2(a)). CHIR99021 produced a slight enhancement of* Mesp1* and* brachyury* transcription, but the increases observed in response to this drug were far less than obtained with BIX01294 (Figure 2(a)). None of the other pharmacological reagents significantly enhanced* Mesp1* and* brachyury* expression over nontreated control levels (Figure 2(a)). Dose response analysis determined that the effective BIX01294 concentration for stimulating precardiac gene expression ranged from 4–12 *μ*M, with optimum dose being 8 *μ*M (Figure 2(b)). Time course analysis demonstrated that 48 hrs of treatment with BIX01294 was optimal for maximizing induction of precardiac gene expression by BIX01294 (Figure 2(c)). Exposure to BIX01294 for longer periods of time was not beneficial and generated reduced levels of precardiac gene expression as compared to two-day treatments.

**Figure 3:**
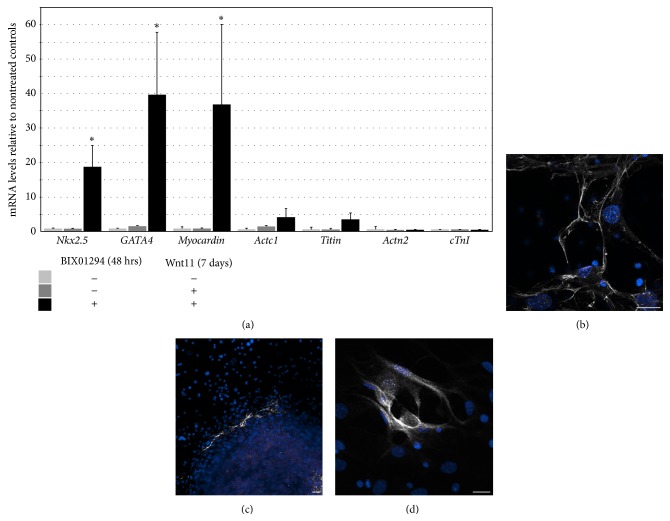
BIX01294 promotes responsiveness to the cardiogenic stimulating protein Wnt11. MSCs were cultured nontreated or with 8 *μ*M BIX01294 for 48 hrs prior to seven-day culture in fresh media with or without Wnt11. (a) Real-time qPCR analysis indicated that MSCs displayed significant upregulation of the primary cardiac transcription factors* Nkx2.5*,* GATA4*, and* myocardin* in response to Wnt11 when pretreated with BIX01294, as compared to cultures that were unexposed to BIX01294. In contrast, genes encoding for* cardiac α-actin* (*Actc1*),* titin*,* muscle α-actinin* (*Actn2*), and* cTnI* showed minimal to nondetectable increases in expression in response to BIX01294 plus Wnt11 treatments. ^*∗*^ *p* < 0.01. (b–d) However, immunofluorescent analysis did demonstrate that BIX01294 plus Wnt11 treatments promoted expression of muscle proteins (gray staining), as shown for (b) desmin and (c, d) *α*-actinin. Low magnification view of *α*-actinin in panel (c) illustrates that expression of muscle proteins was only displayed within small patches of cells as indicated by nuclear counterstaining with DAPI (blue). Scale bar = 20 *μ*m.

After demonstrating that BIX01294 promotes precardiac gene expression, we ascertained whether this treatment would enhance the ability of MSCs to undergo myocardiogenic differentiation. MSCs were cultured in the absence or presence of 8 *μ*M BIX01294 for 48 hrs, prior to subsequent incubation with the cardiac differentiation stimulating factor Wnt11. Following culture with WNT11, cells were isolated and analyzed for cardiac gene expression by qPCR. Cultures without preexposure to BIX01294 displayed only minimal expression of cardiac genes whether or not they were subsequently treated with Wnt11 (Figure 3(a)). In contrast, cultures preincubated with BIX01294 displayed a robust response to Wnt11 by expression of the cardiac transcription factors* Nkx2.5*,* GATA4*, and* myocardin* that was amplified 19, 40, and 37-fold over control cultures (Figure 3(a)). However, transcription of genes encoding for sarcomeric proteins showed little enhancement over control levels (Figure 3(a)). Despite this latter result, immunostaining revealed that, unlike control cultures, BIX01294 plus Wnt11-treated cultures displayed small patches of cells that were positive for cardiac muscle protein expression (Figures 3(b)–3(d)).

### 3.3. BIX01294 Treatment Enhances Formation of Phase Bright Cells from Bone Marrow MSCs

**Figure 4:**
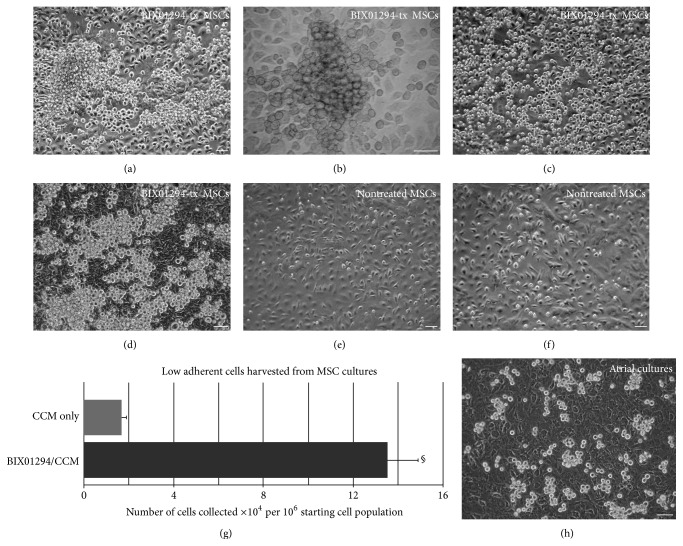
Acquisition of phase bright phenotype by BIX01294-treated MSCs. Phase-contrast images showing (a–d) BIX01294-treated MSCs that were subsequently incubated with CCM. By 48-hour exposure to CCM, BIX01294-treated MSCs (a) exhibited bursts of PBCs (asterisk), (b) which imaged in bright field at higher magnification appear as colony bursts. Subsequently, PBCs derived from BIX01294-treated MSCs spread throughout the cultures, as shown in the following (c) 4 days and (d) 2 weeks of CCM incubation. (e, f) Parallel MSC cultures incubated with CCM without prior treatment with BIX01294 displayed far fewer PBCs, as shown at 2 and 4 days of incubation, respectively. (g) Tabulation of PBC yields from MSC cultures pretreated in presence or absence of BIX01294, as estimated by counting low adherent cells released by brief EDTA exposure. Note that preexposure to BIX01294 generated significantly greater numbers of PBCs from MSC cultures when exposed to CCM (^§^ *p* < 0.001). The display of PBCs from the BIX01294 plus CCM-treated MSC cultures was similar to that (h) produced by cultures of atrial tissue.

**Figure 5:**
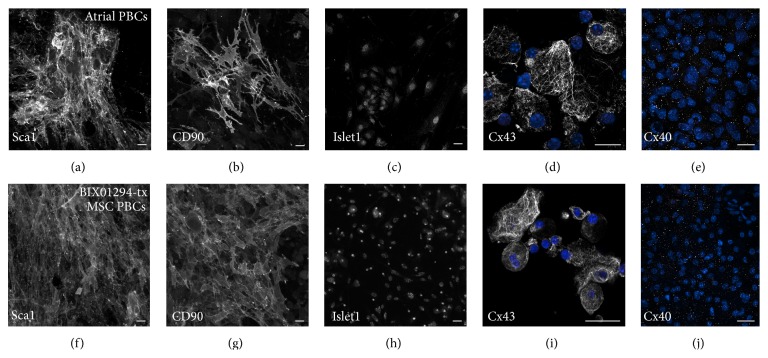
Shared expression of progenitor markers in atrial and bone marrow-derived PBCs. PBCs were collected from either (a–e) atrial cultures or (f–j) BIX01294 plus CCM-treated MSCs and immunostained following (a–c; e–h, i) their attachment on Cell-Tak coated chamber slides or (d, i) being cytospun onto glass slides. Subsequent immunofluorescent labeling of the cells (shown in gray) was used to detect the expression of (a, f) Sca-1 (b, g) CD90 (c, h) Isl-1, (d, i) Cx43, and (e, j) Cx40. Cultures were counterstained with DAPI to visualize nuclei (blue). Scale bar = 25 *μ*m.

The cardiac capacity of MSCs was further tested by placing the cells in a cardiac environment. For these experiments, culture supernatants from adult mouse atrial cultures were used as a source of cardiac conditioned media (CCM) for treating MSCs that were preincubated for 48 hrs in the presence or absence of BIX01294. Within 48 hrs of exposure to CCM, BIX01294-treated MSC cultures began to exhibit bursts of small, round phase bright cells (PBCs) that emerged above the adherent cell layer (Figures 4(a) and 4(b)). During subsequent days of incubation in CCM, PBCs from BIX01294-pretreated cultures spread throughout the culture dish and were densely displayed on top of the remaining adherent cells (Figures 4(c) and 4(d)). Parallel cultures of MSCs incubated with CCM without pretreatment with BIX01294 did not display a similar burst of PBC formation, although small numbers of PBCs did emerge from these control cultures over time (Figures 4(e) and 4(f)). The yields of bone marrow-derived PBCs were estimated by counting low adherent cells released by brief EDTA exposure from cultures collected after 2-week incubation period with CCM. On average, from a starting cell population of 10^6^ cells, the CCM incubation generated 13.5 × 10^4^ and 1.7 × 10^4^ low adherent cells, respectively, from MSCs pretreated in the presence or absence of BIX01294 (Figure 4(g)). PBCs generated in the BIX01294 plus CCM-treated MSC cultures appeared to be morphologically similar to PBCs that arise spontaneously from atrial cultures (Figure 4(h)). Further characterization of PBCs harvested from atrial and BIX01294 + CCM-treated MSC cultures indicated that the similarities between these two populations were not just morphological. Immunofluorescent staining analysis demonstrated that atrial and MSC-derived PBCs shared expression of the stem cell markers Sca1, CD90, and Islet1 (Figure 5). In addition, both PBC cell populations were positive for the gap junction proteins connexin43 (Figures 5(d) and 5(i)) and connexin40 (Figures 5(e) and 5(j)), the latter being detected in both populations only when allowed to form cell-cell contacts.

### 3.4. PBCs Derived from BIX01294-Treated MSCs Are Cardiac Competent

**Figure 6:**
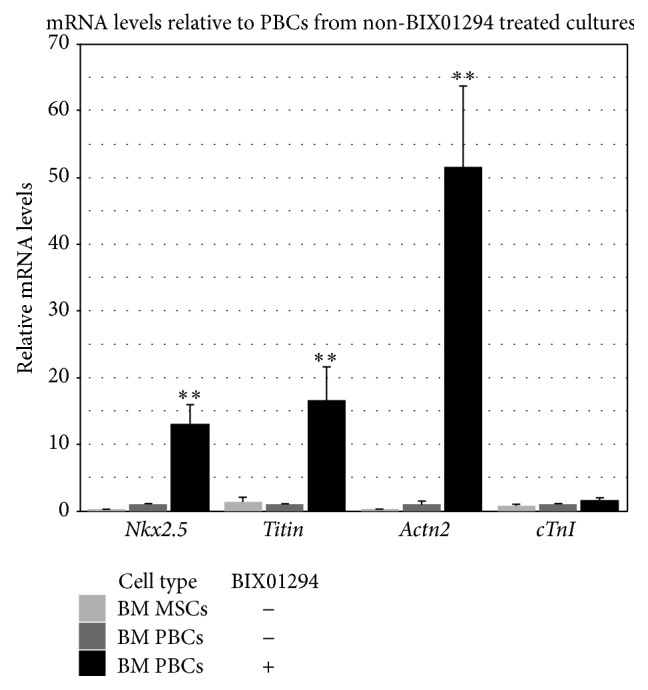
Cardiac differentiation of bone marrow-derived PBCs. PBCs collected from MSC cultures pretreated with or without BIX01294 were aggregated by hanging drop for 4 days and then differentiated in 50% CCM following their transfer to tissue culture plastic. Thereafter, RNA was isolated from these cultures and analyzed for cardiac gene expression using real-time qPCR. As a comparative control, nontreated MSCs were also assayed by qPCR. As shown, BIX01294 exposure produced PBCs that exhibited significant upregulation of the early cardiac markers* Nkx2.5*,* titin*, and* Actn2*, as compared to PBCs obtained from BIX01294-negative cultures. In contrast, expression of the late cardiac marker* cTnI* was not stimulated, which indicates the conditions used in these assays were not sufficient to support complete myocyte differentiation. ^*∗∗*^ *p* < 0.005.

**Figure 7:**
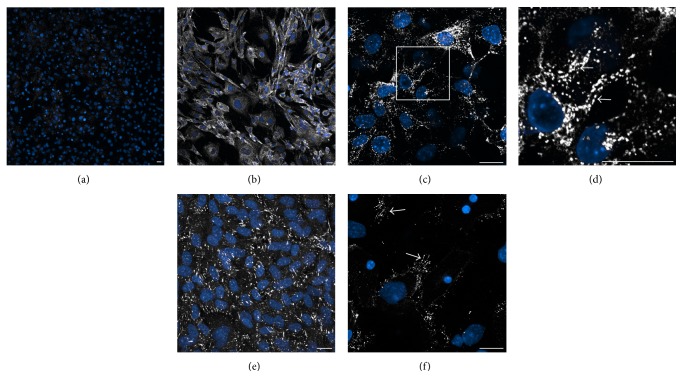
Sarcomeric protein expression of bone marrow-derived PBCs. (a) MSCs cultured with CCM but unexposed to BIX01294 or (b–f) PBCs collected from BIX01294 plus CCM-treated MSCs were subsequently treated with dexamethasone and aggregated as a hanging drop prior to dual fluorescent staining with antibody (gray) and DAPI (blue). (a) Individual control culture showing titin and DAPI staining. Note the absence of gray signal indicating that non-BIX01294-treated control cultures were not labeled with the titin antibody. (b) In contrast, BIX01294 induced PBCs showed widespread titin expression when placed under differentiation conditions. (c) High magnification view of aggregated PBC cultures stained for titin, which on close-up view of the boxed region (d) indicated that titin protein was becoming organized within the cells (arrows). (e) Muscle *α*-actinin was also broadly distributed in MSC-derived PBC cultures, (f) which at higher magnification showed evidence of becoming organized (arrows). Scale bar = 20 *μ*m.

To determine whether bone marrow-derived PBCs were capable of undergoing cardiac differentiation, we examined culture conditions for promoting a myocardial phenotype. PBCs generated from MSCs that were pretreated in the presence or absence of BIX01294 were aggregated by hanging drop for 4 days, prior to being transferred to tissue culture plastic and subsequently incubated in the presence of CCM. Cultures were harvested for RNA and examined by qPCR for transcription of myocardial genes (Figure 6) or immunofluorescently stained as a direct assay for the expression of sarcomeric proteins (Figure 7). Gene expression analysis revealed that BIX01294 pretreatment of MSCs produced PBCs that exhibited a significant enhancement of* Nkx2.5*, muscle *α-actinin* (*Actn2*), and* titin* gene expression, which was 13-, 17-, and 52-fold higher, respectively, than PBCs harvested from parallel cultures unexposed to the G9a HMTase inhibitor (Figure 6). In contrast to these early cardiomyocyte genes, BIX01294 generated PBCs did not exhibit the late myocardial marker* cardiac troponin I* (*cTnI*), when cultured under these conditions (Figure 6). Immunofluorescent staining of parallel cultures demonstrated correlative results at the protein level, as *α*-actinin and titin were displayed throughout the PBC cultures derived from BIX01294-treated MSCs but were absent from BM PBCs cultures that were unexposed to BIX01294 (Figure 7). Moreover, high magnification views of the BIX01294/CCM-generated PBC cultures indicated that these sarcomeric proteins were starting to be displayed in an organized pattern (Figures 7(d) and 7(f)). Collectively, these data suggest MSCs exposed to BIX01294 and subsequently incubated in CCM generated cells exhibiting both a phenotype and differentiation potential resembling endogenous cardiac progenitor cells.

## 4. Discussion

Bone marrow provides an accessible and abundant source of stem cells, which has long been investigated for use in cardiac repair [8–11]. However, both animal studies and clinical trials have produced inconsistent results and, according to some published studies, inauspicious outcomes [8, 45–47]. The present study extends our previous efforts to broaden the phenotypic potential of bone marrow stem cells to enhance their cardiac competency. The development of genetic and nongenetic methods for generating iPSCs demonstrated that the differentiation potential of adult somatic cells could be broadened to produce a pluripotent phenotype [34, 48, 49]. Our goal with bone marrow stem cells was not to produce pluripotent cells, but to develop methods that would broaden their differentiation potential enough to acquire a cardiac competent phenotype, while stopping short of making the cells pluripotent. Since both bone marrow and heart are mesoderm derivatives, we hypothesized that the phenotypic potential of bone marrow stem cells could be broadened to a cell that was pan-mesodermal and therefore cardiocompetent. We screened bone marrow stem cells for responses to a variety of molecules that have been shown to assist the production of iPSCs when combined with other treatments, but are unable by themselves to promote pluripotency. Of the molecules we screened, the only one that appreciably upregulated precardiac markers and allowed bone marrow cells to respond to cardiogenic signals was the G9a HMTase inhibitor BIX01294.

In a previous study, we demonstrated that exposure of bone marrow cells to BIX01294 provokes a pan-mesodermal potential but does not confer competency for endodermal and ectodermal lineages [19]. This report provides evidence confirming that G9a HMTase inhibition confers cardiac competency on nondifferentiated bone marrow cells, as specific knockdown of this gene induces expression of precardiac progenitor cell markers. Transient pharmacological inhibition of G9a HMTase with BIX01294 allowed bone marrow MSCs to respond to cardiogenic stimuli, such as Wnt11, as indicated by an upregulation of primary cardiac transcription factors* Nkx2.5*,* GATA4*, and* myocardin*. The BIX01294/Wnt11 treatments also generated small patches of myofibrillar protein-positive cells, although the expression of these muscle molecules was not widespread enough to be reflected in the overall levels of gene expression of the entire cultures. When MSCs pretreated with BIX01294 were subsequently incubated with CCM, the cultures generated PBCs that exhibited morphological and molecular characteristics resembling cells that arise spontaneously from atrial cultures. Atrial PBCs, also referred to as cardiosphere-forming cells, are thought to comprise the endogenous cardiac progenitor cells that contribute to myocyte renewal in the adult heart [4–7]. Like endogenous cardiac progenitor cells, bone marrow-derived PBCs that arose from BIX01294 pretreatment and incubation with CCM showed evidence that they can differentiate to cardiac myocytes. When these PBCs were aggregated as a hanging drop and further incubated with CCM, the bone marrow-derived cells showed significant enhancement in expression of *α*-actinin and titin, which are two of the primary sarcomeric proteins exhibited by differentiating cardiomyocytes of the early embryo [50, 51]. While the absence of the late cardiac marker cTnI [52, 53] indicates that the hanging drop assay did not generate fully differentiated myocytes, the burgeoning striations observed for *α*-actinin and titin suggests that myofibrillogenesis was being initiated in the bone marrow-derived PBCs. MSCs cultured without BIX01294 pretreatment also produced PBCs when incubated with CCM. Yet, the yields were far less than exhibited by BIX01294 pretreated cultures. Unlike PBCs derived from BIX01294-treated MSCs, PBCs from cultures unexposed to BIX01294 did not show evidence of cardiomyogenesis. Thus, the key component in promoting cardiocompetency from bone marrow cells was BIX01294, as cells cultured under identical conditions, but without preexposure to this reagent, did not display sarcomeric protein expression.

BIX01294 has been shown to be highly specific against G9a HMTase [30, 32], although that does not necessarily rule out that the efficacy of this drug in fostering a cardiac competent phenotype from bone marrow cells is due to its side effects on other uncharacterized targets. However, the corresponding outcomes observed in response to BIX01294 treatment and G9a HMTase knockdown would appear to indicate that the influence BIX01294 has on cell phenotypic potential is due to its inhibition of this enzyme. In addition, there was correlation between the overall decrease in H3K9 methylation and the effect of G9a HMTase knockdown or BIX01294 exposure in promoting a change in bone marrow cell phenotype. Studies examining the activity of G9a HMTase have indicated that lysine 9 on histone H3 is a principal substrate of this enzyme although lysine 27 on histone H3 has been identified as an additional target [20, 32, 33, 54]. Recent studies have suggested that G9a HMTase may also modify several nonhistone proteins [55, 56] that our present data does not preclude as candidate molecular targets responsible for conferring cardiac competency to bone marrow cells. Yet, when the extensive characterization of this enzyme's histone regulatory activities and associated effects on gene transcription are considered, it seems likely that the methylation state of lysine 9 and possibly lysine 27 on histone H3 are key determinants in influencing whether a stem cell exhibits cardiac potential.

Chromatin remodeling during differentiation is thought to play a critical role in restricting cell phenotype. Dimethylation of histone H3 by G9a HMTase is associated with transcriptional repression [20, 56, 57]. It has been postulated that the utility of BIX01294 for assisting the generation of iPS cells is due to its ability to reverse transcriptional repression of stem cell genes [34, 48, 49]. While these properties allow BIX01294 to help confer a pluripotent phenotype from adult somatic cells, it must be accompanied by other pharmacological and/or genetic manipulations. Our studies would suggest that the effects mediated on chromatin structure and gene expression by BIX01294 are sufficient on their own to broaden the phenotypic potential of bone marrow MSCs enough to make cells competent to generate cardiac myocytes and possibly cells of other mesodermal lineages.

In summary, we report that bone marrow MSCs can be made cardiac competent by transient exposure to the G9a HMTase inhibitor BIX01294. Expressions of the early mesodermal, precardiac markers* Mesp1* and* brachyury* in response to BIX01294 exposure suggest that the MSCs have been converted to pan-mesodermal, cardiocompetent cell phenotype. Subsequent incubation of BIX01294-treated MSCs with CCM prompts the formation of PBCs that resemble endogenous cardiac progenitors and have an increased capacity to undergo cardiac differentiation. Collectively, these data suggest that BIX01294 has utility as a pharmacological component of protocols that could be used to enhance the ability of an abundant and accessible stem cell population for cardiac repair. Future experimentation examining how BIX01294-treated MSCs compare to endogenous cardiac progenitor cells in animal transplantation assays and whether G9a HMTase inhibition has similar effects on the cardiac potential of adult human stem cells will test the hypotheses suggested by the present study.

## References

[1] Beltrami A. P., Barlucchi L., Torella D. (2003). Adult cardiac stem cells are multipotent and support myocardial regeneration. *Cell*.

[2] Hsieh P. C. H., Segers V. F. M., Davis M. E. (2007). Evidence from a genetic fate-mapping study that stem cells refresh adult mammalian cardiomyocytes after injury. *Nature Medicine*.

[3] Nadal-Ginard B., Ellison G. M., Torella D. (2014). The cardiac stem cell compartment is indispensable for myocardial cell homeostasis, repair and regeneration in the adult. *Stem Cell Research*.

[4] Chan H. H. L., Meher Homji Z., Gomes R. S. M. (2012). Human cardiosphere-derived cells from patients with chronic ischaemic heart disease can be routinely expanded from atrial but not epicardial ventricular biopsies. *Journal of Cardiovascular Translational Research*.

[5] Davis D. R., Zhang Y., Smith R. R. (2009). Validation of the cardiosphere method to culture cardiac progenitor cells from myocardial tissue. *PLoS ONE*.

[6] Messina E., de Angelis L., Frati G. (2004). Isolation and expansion of adult cardiac stem cells from human and murine heart. *Circulation Research*.

[7] Ye J., Boyle A., Shih H. (2012). Sca-1^+^ cardiosphere-derived cells are enriched for isl1-expressing cardiac precursors and improve cardiac function after myocardial injury. *PLoS ONE*.

[8] Dai W., Kloner R. A. (2011). Bone marrow-derived cell transplantation therapy for myocardial infarction: lessons learned and future questions. *American Journal of Transplantation*.

[9] Eisenberg L. M., Eisenberg C. A. (2004). Adult stem cells and their cardiac potential. *Anatomical Record Part A: Discoveries in Molecular, Cellular, and Evolutionary Biology*.

[10] Kuçi S., Kuçi Z., Latifi-Pupovci H. (2009). Adult stem cells as an alternative source of multipotential (pluripotential) cells in regenerative medicine. *Current Stem Cell Research & Therapy*.

[11] Rota M., Kajstura J., Hosoda T. (2007). Bone marrow cells adopt the cardiomyogenic fate in vivo. *Proceedings of the National Academy of Sciences of the United States of America*.

[12] Chow M. Z., Boheler K. R., Li R. A. (2013). Human pluripotent stem cell-derived cardiomyocytes for heart regeneration, drug discovery and disease modeling: from the genetic, epigenetic, and tissue modeling perspectives. *Stem Cell Research & Therapy*.

[13] Lalit P. A., Hei D. J., Raval A. N., Kamp T. J. (2014). Induced pluripotent stem cells for post-myocardial infarction repair: remarkable opportunities and challenges. *Circulation Research*.

[14] Menasché P., Vanneaux V., Fabreguettes J. R. (2014). Towards a clinical use of human embryonic stem cell-derived cardiac progenitors: a translational experience. *European Heart Journal*.

[15] Yu T., Miyagawa S., Miki K. (2013). In vivo differentiation of induced pluripotent stem cell-derived cardiomyocytes. *Circulation Journal*.

[16] Anderson M. E., Goldhaber J., Houser S. R. (2014). Embryonic stem cell-derived cardiac myocytes are not ready for human trials. *Circulation Research*.

[17] Fong C.-Y., Gauthaman K., Bongso A. (2010). Teratomas from pluripotent stem cells: a clinical hurdle. *Journal of Cellular Biochemistry*.

[18] Wobus A. M. (2010). The Janus face of pluripotent stem cells—connection between pluripotency and tumourigenicity. *BioEssays*.

[19] Mezentseva N. V., Yang J., Kaur K. (2013). The histone methyltransferase inhibitor BIX01294 enhances the cardiac potential of bone marrow cells. *Stem Cells and Development*.

[20] Tachibana M., Sugimoto K., Nozaki M. (2002). G9a histone methyltransferase plays a dominant role in euchromatic histone H3 lysine 9 methylation and is essential for early embryogenesis. *Genes & Development*.

[21] Chamberlain G., Fox J., Ashton B., Middleton J. (2007). Concise review: mesenchymal stem cells: their phenotype, differentiation capacity, immunological features, and potential for homing. *Stem Cells*.

[22] Soleimani M., Nadri S. (2009). A protocol for isolation and culture of mesenchymal stem cells from mouse bone marrow. *Nature Protocols*.

[23] Xu S., De Becker A., Van Camp B., Vanderkerken K., Van Riet I. (2010). An improved harvest and in vitro expansion protocol for murine bone marrow-derived mesenchymal stem cells. *Journal of Biomedicine and Biotechnology*.

[24] Eisenberg C. A., Eisenberg L. M. (1999). WNT11 promotes cardiac tissue formation of early mesoderm. *Developmental Dynamics*.

[25] Eisenberg C. A., Burch J. B. E., Eisenberg L. M. (2006). Bone marrow cells transdifferentiate to cardiomyocytes when introduced into the embryonic heart. *Stem Cells*.

[26] Eisenberg L. M., Burns L., Eisenberg C. A. (2003). Hematopoietic cells from bone marrow have the potential to differentiate into cardiomyocytes *in vitro*. *The Anatomical Record Part A: Discoveries in Molecular, Cellular, and Evolutionary Biology*.

[27] Martin L. K., Mezentseva N. V., Bratoeva M., Ramsdell A. F., Eisenberg C. A., Eisenberg L. M. (2011). Canonical WNT signaling enhances stem cell expression in the developing heart without a corresponding inhibition of cardiogenic differentiation. *Stem Cells and Development*.

[28] Martin L. K., Bratoeva M., Mezentseva N. V. (2012). Inhibition of heart formation by lithium is an indirect result of the disruption of tissue organization within the embryo. *Development Growth and Differentiation*.

[29] Rémond M. C., Iaffaldano G., O'Quinn M. P. (2011). GATA6 reporter gene reveals myocardial phenotypic heterogeneity that is related to variations in gap junction coupling. *American Journal of Physiology—Heart and Circulatory Physiology*.

[30] Chang Y., Zhang X., Horton J. R. (2009). Structural basis for G9a-like protein lysine methyltransferase inhibition by BIX-01294. *Nature Structural and Molecular Biology*.

[31] Imai K., Togami H., Okamoto T. (2010). Involvement of histone H3 lysine 9 (H3K9) methyltransferase G9a in the maintenance of HIV-1 latency and its reactivation by BIX01294. *The Journal of Biological Chemistry*.

[32] Kubicek S., O'Sullivan R. J., August E. M. (2007). Reversal of H3K9me2 by a small-molecule inhibitor for the G9a histone methyltransferase. *Molecular Cell*.

[33] Chen H., Yan Y., Davidson T. L., Shinkai Y., Costa M. (2006). Hypoxic stress induces dimethylated histone H3 lysine 9 through histone methyltransferase G9a in mammalian cells. *Cancer Research*.

[34] Feng B., Ng J. H., Heng J. C. D., Ng H. H. (2009). Molecules that promote or enhance reprogramming of somatic cells to induced pluripotent stem cells. *Cell Stem Cell*.

[35] Huangfu D., Maehr R., Guo W. (2008). Induction of pluripotent stem cells by defined factors is greatly improved by small-molecule compounds. *Nature Biotechnology*.

[36] Ichida J. K., Blanchard J., Lam K. (2009). A small-molecule inhibitor of Tgf-*β* signaling replaces sox2 in reprogramming by inducing nanog. *Cell Stem Cell*.

[37] Mora-Castilla S., Tejedo J. R., Hmadcha A. (2010). Nitric oxide repression of Nanog promotes mouse embryonic stem cell differentiation. *Cell Death and Differentiation*.

[38] Silva J., Barrandon O., Nichols J., Kawaguchi J., Theunissen T. W., Smith A. (2008). Promotion of reprogramming to ground state pluripotency by signal inhibition. *PLoS Biology*.

[39] Yao K., Ki M. O., Chen H. (2014). JNK1 and 2 play a negative role in reprogramming to pluripotent stem cells by suppressing Klf4 activity. *Stem Cell Research*.

[40] Ying Q.-L., Wray J., Nichols J. (2008). The ground state of embryonic stem cell self-renewal. *Nature*.

[41] Bondue A., Blanpain C. (2010). Mesp1: a key regulator of cardiovascular lineage commitment. *Circulation Research*.

[42] David R., Jarsch V. B., Schwarz F. (2011). Induction of MesP1 by Brachyury(T) generates the common multipotent cardiovascular stem cell. *Cardiovascular Research*.

[43] Kattman S. J., Huber T. L., Keller G. M. (2006). Multipotent flk-1^+^ cardiovascular progenitor cells give rise to the cardiomyocyte, endothelial, and vascular smooth muscle lineages. *Developmental Cell*.

[44] Wu S. M. (2008). Mesp1 at the heart of mesoderm lineage specification. *Cell Stem Cell*.

[45] Goumans M. J., Maring J. A., Smits A. M. (2014). A straightforward guide to the basic science behind cardiovascular cell-based therapies. *Heart*.

[46] Pfister O., Della Verde G., Liao R., Kuster G. M. (2014). Regenerative therapy for cardiovascular disease. *Translational Research*.

[47] Wysoczynski M., Hong K. U., Moore J. B. (2014). Bone marrow cell therapies in ischemic cardiomyopathy. *Expert Opinion on Biological Therapy*.

[48] Hayes M., Zavazava N. (2013). Strategies to generate induced pluripotent stem cells. *Methods in Molecular Biology*.

[49] Hou P., Li Y., Zhang X. (2013). Pluripotent stem cells induced from mouse somatic cells by small-molecule compounds. *Science*.

[50] Tokuyasu K. T., Maher P. A. (1987). Immunocytochemical studies of cardiac myofibrillogenesis in early chick embryos. I. Presence of immunofluorescent titin spots in premyofibril stages. *The Journal of Cell Biology*.

[51] Tokuyasu K. T., Maher P. A. (1987). Immunocytochemical studies of cardiac myofibrillogenesis in early chick embryos. II. Generation of *α*-actinin dots within titin spots at the time of the first myofibril formation. *Journal of Cell Biology*.

[52] Ausoni S., de Nardi C., Moretti P., Gorza L., Schiaffino S. (1991). Developmental expression of rat cardiac troponin I mRNA. *Development*.

[53] Martin A. F., Orlowski J. (1991). Molecular cloning and developmental expression of the rat cardiac-specific isoform of troponin I. *Journal of Molecular and Cellular Cardiology*.

[54] Wu H., Chen X., Xiong J. (2011). Histone methyltransferase G9a contributes to H3K27 methylation in vivo. *Cell Research*.

[55] Moore K. E., Carlson S. M., Camp N. D. (2013). A general molecular affinity strategy for global detection and proteomic analysis of lysine methylation. *Molecular Cell*.

[56] Shinkai Y., Tachibana M. (2011). H3K9 methyltransferase G9a and the related molecule GLP. *Genes & Development*.

[57] Epsztejn-Litman S., Feldman N., Abu-Remaileh M. (2008). De novo DNA methylation promoted by G9a prevents reprogramming of embryonically silenced genes. *Nature Structural and Molecular Biology*.

